# Success in Medical Practice

**Published:** 1874-11

**Authors:** 


Success in Medical Practice.—Many young men are now about to enter upon the study of medicine, full of hopes and fears, and above all things anxious to find out and use the proper means for insuring a prosperous career. It should be known to them that the factors which contribute to the formation of a successful medical man are very numerous. These may be divided into scientific and social. It is quite possible for a man to acquire considerable eminence either from his scientific attainments or from his social qualities; but the highest position is only to be reached by the possession of all the qualifications comprehended under both heads. At the onset, however, it must be admitted that not every one can gain this exalted status. It behooves, therefore, each student carefully to examine himself from time to time, and endeavor to discover wherein lies his weakness and his strength, for in this way alone can he find out in what branch of medical practice he is most likely to succeed, and to what position in his profession he may aspire. It may be broadly stated that all who are able to pass the preliminary examination can by careful study possess themselves of the elementary knowledge demanded before they can become legally qualified practitioners. Those whose natural abilities and means enable them to go further than this, and who determine to run a higher and harder race, must, when they have decided what their goal shall be, keep it persistently in sight, and move on towards it with unswerving purpose. The first step towards success is to obtain in as simple and effective a way as possible, a thoroughly systematic knowledge of all subjects connected with medicine ; and by this is meant not simply a memorial mastery, but a complete mental digestion and assimilation of medical science—not blindly following writers without verifying their references, nor accepting the dicta of authorities without repeating their experiments. This in itself is a herculean labor, for, although begun during student life, it has to be carried on continuously. Nevertheless, thus alone can the pinnacle of medical eminence be mounted. The social qualifications which conduce to success in medical practice are very many. Health is most important, for without it no one can, year after year, bear continuous bodily fatigue and mental strain. Endurance is absolutely necessary. It enables its owner to bear with the whims of patients and annoyances of practice, and supports him in the still more difficult task of waiting till patients and practice arrive. A sympathizing and gentle manner always wins staunch friends. Accomplishments tend to popularity. A good writer, speaker, or linguist has immense advantages, as also has a man of refinement, well dressed, with a fine person and good looks. Nerve and tact are indispensable, and a strict observance of social and medical ethics is essentially necessary. These qualifications act beneficially, and with equal force, whatever branch of the profession may be chosen. Special practice, however, demands special aptitude. Almost all men have a natural predilection for one particular department of their profession. This often displays itself early, and continues steadily through life, even spite of the distractions of general practice. It is good to encourage this liking, for even should it not end in its possessor becoming a special practitioner, it afterwards assists materially in keeping up an interest in his profession, in lightening his labors, and extending his knowledge. The disposition to practice specially can only be indulged in large towns, and those who adopt it often succeed wonderfully and enjoy a highly honorable position. The division which embraces the largest amount of work is, without doubt, Obstetric medicine. Midwifery and the diseases of women and children form two-thirds of the occupation of general practitioners. It is, therefore, fortunate when a student takes interest in this great branch, for more than any other it is likely to open up to him a prosperous path. Let him not be misled by the inadequate means at present existing for teaching it into the belief that it is of little importance. If he has a strong liking for it, or for any other department, let him, if possible, follow fearlessly his inclination, resting most certainly assured, whatever line he may adopt, that honest work and righteous life will win respect and lead unerringly to success in practice.—*The Obstetrical Journal.*

